# Brain lesions in pediatric abusive head trauma: prevalence, pathophysiology, patterns, and a classification system

**DOI:** 10.1007/s00330-025-11895-5

**Published:** 2025-08-14

**Authors:** Maria Hahnemann, Bernd Karger, Heidi Pfeiffer, Hans-Joachim Mentzel, Alexander Radbruch, Daniel Wittschieber

**Affiliations:** 1 Department of Diagnostic and Interventional Neuroradiology and Pediatric Neuroradiology, University Hospital Bonn, University of Bonn, Venusberg-Campus 1, 53127 Bonn, Germany; 2 Institute of Legal Medicine, University Hospital Münster, University of Münster, Röntgenstraße 23, 48149 Münster, Germany; 3 Section of Pediatric Radiology, Department of Radiology, Jena University Hospital, University of Jena, Am Klinikum 1, 07747 Jena, Germany; 4 Institute of Legal Medicine, University Hospital Bonn, University of Bonn, Stiftsplatz 12, 53111 Bonn, Germany

**Keywords:** Child physical abuse, Non-accidental head injury, Traumatic brain injury, Hypoxic-ischemic injury, Cytotoxic brain edema

## Abstract

**Objectives:**

A deeper understanding of extensive brain lesions (EBL) in pediatric abusive head trauma (AHT) could possibly help differentiate AHT from other forms of trauma. Therefore, the aims of the study were (i) to investigate the prevalence and features of AHT-associated EBL in neuroimaging and (ii) to develop a useful classification system.

**Materials and methods:**

This retrospective multicenter study analyzed cranial CT and/or MRI of medico-legally well-documented AHT cases diagnosed with “shaken baby syndrome” from a 10-year study period with respect to lesions in cerebrum, cerebellum, and brain stem. For the development of a classification system, EBLs were grouped into distinct lesion patterns based on laterality, symmetry, distribution, and shape.

**Results:**

A total of 61 AHT cases were included. Comparison between “confession cases” (*n* = 15) and “non-confession cases” (*n* = 46) did not show any statistically significant difference regarding all parameters analysed. EBL were found in ~ 1/3 of the cases (*n* = 20). Brain stem lesions were only rarely observed (*n* = 2). Nine different and partly new patterns of EBL occurring in AHT by shaking are described. Pattern analysis revealed that most lesions can be caused by hypoxic-ischemic injury, but evidence is provided that additional pathomechanisms, such as hypoglycemia and the “second impact syndrome”, may be causative or a component.

**Conclusions:**

Brain lesions in AHT by shaking form typical patterns that can be categorized. Considering the rare observation of brain stem lesions, the widespread hypothesis of primary brain stem lesions leading to initial respiratory insufficiency in AHT by shaking should be questioned.

**Key Points:**

***Question*** *Current data on extensive brain lesion patterns in abusive head trauma are insufficient with regard to nomenclature, pathophysiology, and a classification system.*

***Findings*** *Nine different patterns of extensive brain lesions were found in abusive head trauma by shaking, which indicates different pathomechanisms and may help diagnose child abuse.*

***Clinical relevance*** *Consideration of the identified lesion patterns provides new insights into underlying pathomechanisms and supports the clinical diagnosis of abusive head trauma.*

**Graphical Abstract:**

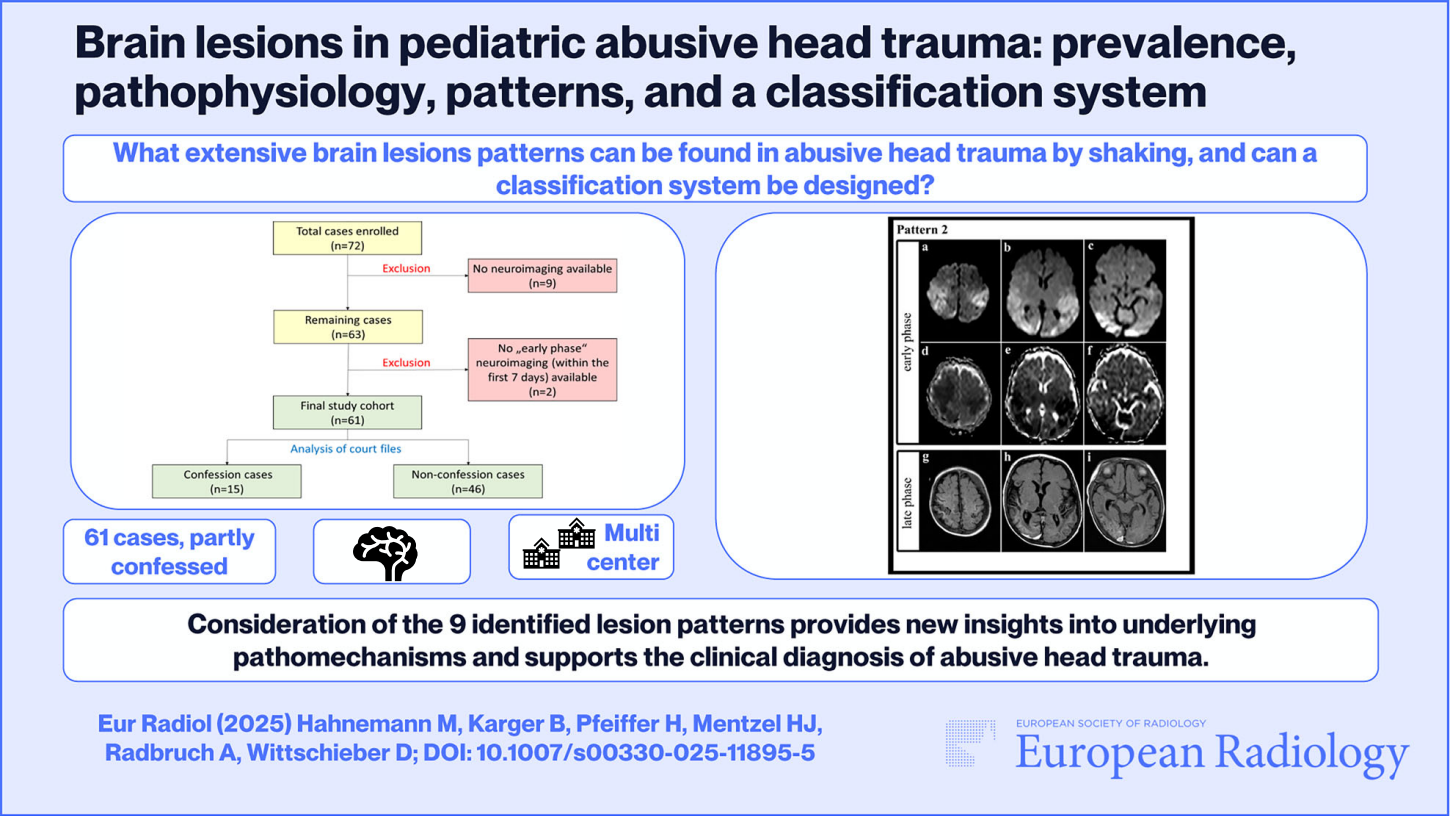

## Introduction

The term “abusive head trauma” (AHT) is used for any inflicted or non-accidental head injuries in pediatrics, whether by forceful shaking, blunt force trauma, or a combination of both [1]. With a probably underestimated incidence of 20–30 in 100,000 live births, AHT is the leading cause of morbidity and mortality in children within their first 2 years of life, suffering from traumatic brain injury [2, 3]. Besides subdural collections, retinal hemorrhages, spinal injuries, and certain types of fractures, lesions of the brain parenchyma are common findings in AHT. The brain injuries are held responsible for severely impairing sequelae, such as developmental delays, hemi- or tetraplegia, epileptic seizures, blindness, deafness, or life-threatening respiratory disorders [4–6].

AHT-associated brain lesions can be subdivided into focal brain lesions (FBL), such as locally restricted contusions and lacerations, and extensive brain lesions (EBL), which can be regarded as widespread brain lesions with parenchymal cytotoxic edema [7]. While direct mechanical injury mechanisms are basically accepted for the formation of FBL, knowledge concerning the nature and etiopathology of EBL is incomplete [8]. Mostly, they are interpreted as (secondary) hypoxic-ischemic injury (HII) [8–12]. In recent years, several different EBL patterns have been described, including predominantly bilateral HII in a distribution consistent with global hypoperfusion [8], diffuse supratentorial brain swelling (infarction) involving the cortex and white matter of all cerebral lobes bilaterally [9], watershed infarction [9], uni- or bilateral hypoxic-ischemic encephalopathy [10], bilateral hypoxic-ischemic pattern [11], asymmetric or symmetric bilateral cytotoxic edema involving both the cortex and the subcortical white matter [12] as well as cytotoxic edema in an extensive bilateral cortical, subcortical, and deep gray matter (DGM) distribution [12]. However, a common nomenclature and a classification system are still lacking [7].

A deeper understanding of the pathophysiology and the neuroimaging appearance of AHT-associated brain lesions could possibly help differentiate AHT from other forms of trauma. This may be valuable for both clinical and forensic specialists as therapeutic approaches could become more targeted and legal procedures more robust. Therefore, the objectives of this study are to investigate the prevalence and features of AHT-associated lesions of the brain parenchyma in cross-sectional neuroimaging (CT and MRI) with special focus on EBL, and to develop a useful classification system for EBL considering anatomy, pathophysiology, and distribution patterns. In particular, compared to previous studies [8–12], methodological emphasis should be placed on a more systematic and detailed analysis of the morphological characteristics of the EBL, with a specific focus on identifying any consistent or emerging patterns.

## Materials and methods

### Data collection

This retrospective, multi-center study was approved by the institutional review boards of all participating institutions, which are the university hospitals of the German cities of Münster, Essen, and Cologne (no. 2014-658-f-N, Medical Association of Westfalen-Lippe and the Westphalian Wilhelms University). Due to the study design, informed consent of the study subjects and their relatives was waived. Other medical and juridical aspects of the present study population (all or part) have recently been investigated and reported in previous publications [13–18].

**Fig. 1:**
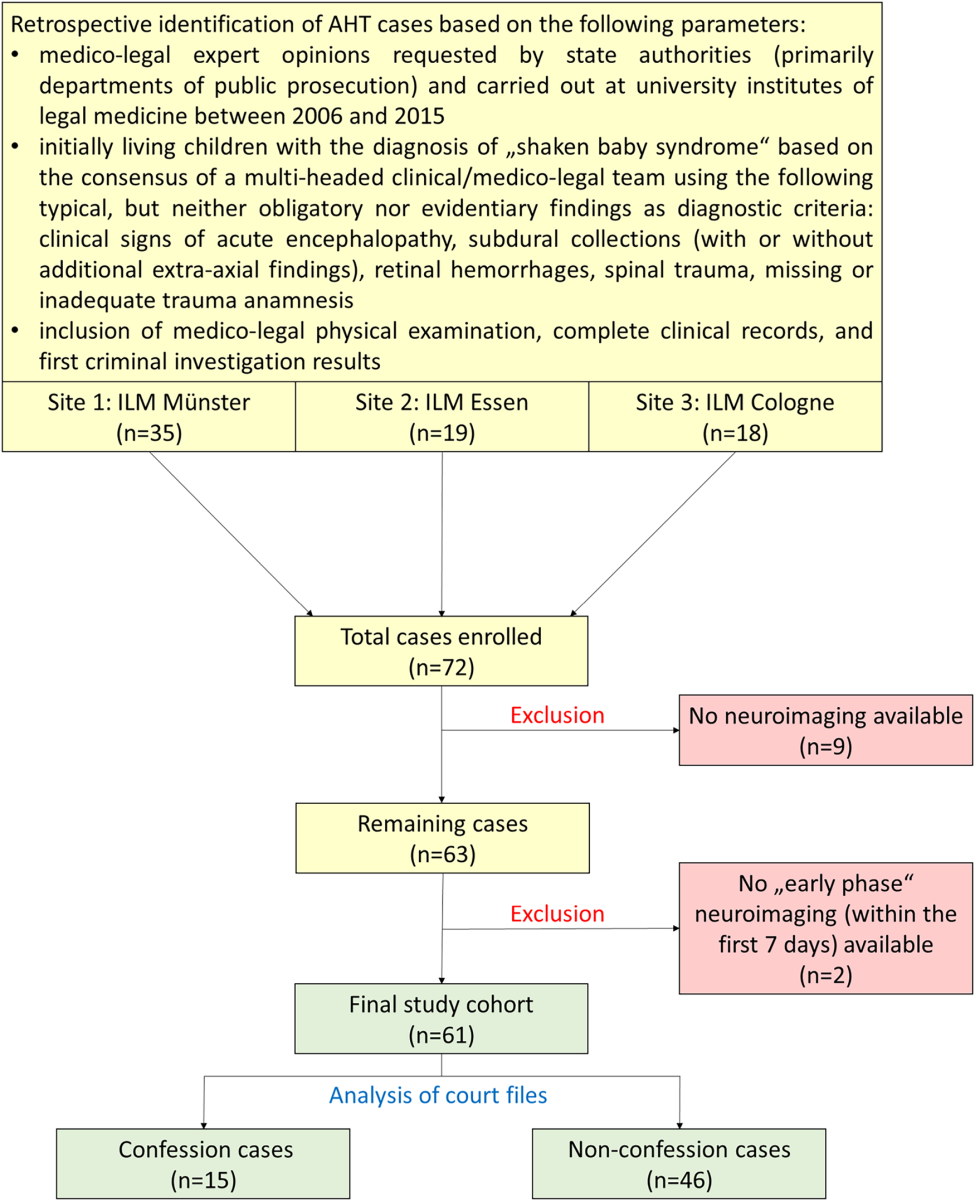
Flow chart demonstrating the process of case selection. AHT, abusive head trauma; ILM, Institute of Legal Medicine

Figure 1 visualizes the process of case selection, including the inclusion and exclusion criteria. The study comprises medico-legally well-documented AHT cases (explicitly diagnosed as “shaken baby syndrome”) from three German university institutes of legal medicine (Münster, Essen, and Cologne) processed between 2006 and 2015 (*n *= 72). Cross-sectional neuroimaging (CT and/or MRI) was available in 63 cases. Similar to previous studies [8, 9, 11, 12], only cases in which neuroimaging was performed within the “early phase” after admission to hospital (≤ 7 days) were included for further analysis (*n* = 61). This time period corresponds to the known detectability of acute brain lesions; for example, due to the sensitivity of diffusion-weighted imaging (DWI) including apparent diffusion coefficient (ADC) analysis for cytotoxic edema over this time span [19, 20]. Accordingly, neuroimaging within the first 7 days of hospitalization was defined as “early phase”, and additional follow-up imaging after the first week (> 7 days) of hospitalization as “late phase”.

Based on previous analyses of the related court files [13], which included perpetrator statements, the final study cohort could be subdivided into “confession cases” (subgroup with confession to violent shaking, *n* = 15) and “non-confession cases” (subgroup without confession to violent shaking, *n* = 46) and compared with each other.

### Neuroimaging protocols

Imaging techniques and protocols varied due to the large time span considered and due to the retrospective multi-center approach. The standard CT and MRI protocols of all cases comprised an appropriate image quality for brain imaging.

The CT examinations were performed on a 16-, 40-, or 256-slice CT scanner using a tube voltage of 120–140 kV, tube current of 40–360 mA, a slice thickness (ST) of 1.5–5 mm, a field of view (FOV) of 150–230 mm, and a matrix of 512 × 512 pixels.

MR imaging was obtained on either a 1.5-T or 3-T scanner. The imaging protocols essentially included T1-weighted (T1w), T2-weighted (T2w), T2w dark fluid, gradient echo (GRE; T2*/susceptibility-weighted imaging, SWI), and diffusion-weighted imaging (DWI) with the following imaging parameters: T1w images had a repetition time (TR) of 244–1000 ms, echo time (TE) of 5–15 ms, ST of 3–6 mm, and a slice gap (SG) of 3.3–6.5 mm. T2w images had a TR/TE of 3000–10,000/81–160 ms, ST of 2–5.5 mm, and a SG of 1–6.5 mm. T2w dark fluid images had a TR/TE of 5700–11,000/80–140 ms, ST of 2–6 mm, and a SG of 1.2–6.6 mm. GRE (T2*/SWI) had a TR/TE of 28–2200/15–40 ms, ST of 1.2–7 mm, and a SG of 1.2–8.4 mm. DWI (*b*-values: b0-b1000 with calculation of an ADC) had a TR/TE of 2900–9200/70–150 ms, ST of 3–6 mm, and a SG of 3.3–7.8 mm. FOV ranged between 150–230 mm. A contrast agent was administered in 19 MRI examinations.

### Image analysis

Image evaluation was jointly performed by one board-certified radiologist and one board-certified forensic physician, each with more than 10 years of specific experience in neuroimaging of child abuse cases. In a few cases with a lack of consensus (*n* < 10), a second board-certified radiologist with more than 15 years of specific experience in pediatric neuroimaging was consulted until consensus was achieved.

In a first step, the “early phase” imaging material was analyzed. The cerebrum was investigated regarding the presence of FBL and EBL. Brain lesions were generally defined as brain areas with signs of (1) edema (e.g., in CT: hypodensity with loss of gray and white matter differentiation; in MRI: hyperintensity in trace image (DWI) and hypointensity in the ADC map), (2) intraparenchymal blood collections (e.g., in CT: hyperdensity; in MRI: positive signal in SWI), or (3) loss of brain tissue. FBL were defined as lesions restricted to one lobe of the brain and/or limited to a maximum of 5 cm. EBLs were defined as widespread lesions expanding over more than one lobe of the brain. For several reasons explained in the discussion section, we decided to use the term “extensive” instead of “diffuse”.

In a second step, the neuroimaging material of the “late phase”, if available, was analyzed for evaluation of potentially missed lesions and lesion development (unchanged, increase/decrease in extent, or disappearance).

In addition, brain lesions within the cerebellum and the brainstem were recorded.

For the development of a classification system, a targeted search was conducted for recurring EBL patterns in our cohort that could be described using only a few typical morphological neuroimaging features. A logical and systematic order for the application of these features was to be followed. Accordingly, we defined four main criteria to be applied in this specific order for the classification of the EBL:

1. Laterality (unilateral/bilateral)
2. Symmetry (symmetrical/asymmetrical)
3. Distribution:
  Brain tissue concerned (e.g., cortico-subcortical (CSC) and/or deep gray matter (DGM) and/or deep white matter (DWM)).
  Brain regions concerned (e.g., frontal, occipital, or along the supply areas of major cerebral arteries).
4. Shape (e.g., spot-like or wedge-shaped).

### Statistical analysis

Statistical data analyses were performed using IBM SPSS Statistics (version 29.0.0.0). Continuous variables were presented as the mean ± standard deviation, median, and range, and compared using *t*-test. Categorical variables were assessed by *χ2* test. *p* < 0.05 was considered statistically significant.

## Results

### Study cohort and neuroimaging material

The main characteristics of the final study cohort are provided in Table 1. The comparison between “confession cases” and “non-confession cases” did not show any statistically significant difference regarding all parameters analyzed (Table 1). The presence of evaluable neuroimaging studies is summarized in Table 2.

**Table 1:** Synopsis of the main characteristics of the study population, including comparison of confession cases and non-confession cases

	Total study cohort	Confession cases	Non-confession cases	Comparison of confession vs. non-confession cases (*p*-value)
***n***	61	15/61 (24.6%)	46/61 (75.4%)	-
**Age in months**				
Mean ± SD	4.3 ± 4.1	2.5 ± 1.3	4.8 ± 4.5	0.057
Median	3	2	3.5	0.200
Range	0–19	1–5	0–19	-
**Sex,*n*** **(%)**				
Male	40/61 (65.6%)	10/15 (66.7%)	30/46 (65.2%)	0.918
Female	21/61 (34.4%)	5/15 (33.3%)	16/46 (34.8%)	0.918
**Main imaging findings,** ***n*** **(%)**				
FBL	15/61 (24.6%)	3/15 (20.0%)	12/46 (26.1%)	0.896
EBL	20/61 (32.8%)	7/15 (46.7%)	13/46 (28.3%)	0.316
FBL only	11/61 (18.0%)	2/15 (13.3%)	9/46 (19.6%)	0.874
EBL only	16/61 (26.2%)	6/15 (40.0%)	10/46 (21.7%)	0.290
**EBL patterns described in Fig. 2,*n*** **(%)**				
Pattern 1	1/61 (1.6%)	0/15 (0%)	1/46 (2.2%)	0.565
Pattern 2	5/61 (8.2%)	1/15 (6.7%)	4/46 (8.7%)	0.804
Pattern 3	5/61 (8.2%)	2/15 (13.3%)	3/46 (6.5%)	0.769
Pattern 4	2/61 (3.3%)	1/15 (6.7%)	1/46 (2.2%)	0.989
Pattern 5	1/61 (1.6%)	1/15 (6.7%)	0/46 (0%)	0.552
Pattern 6	2/61 (3.3%)	1/15 (6.7%)	1/46 (2.2%)	0.989
Pattern 7	2/61 (3.3%)	0/15 (0%)	2/46 (4.3%)	0.412
Pattern 8	1/61 (1.6%)	1/15 (6.7%)	0/46 (0%)	0.552
Pattern 9	1/61 (1.6%)	0/15 (0%)	1/46 (2.2%)	0.565

*n* number of cases, *%* percent, *SD* standard deviation, *FBL* focal brain lesions, *EBL* extensive brain lesions

**Table 2:** Presence of evaluable neuroimaging studies in “early phase” and “late phase”

	*n* (%)
**“Early phase” imaging**	
MRI and/or CT	61/61 (100%)
MRI	57/61 (93.4%)
CT	4/61 (6.6%)
MRI and CT	24/61 (39.3%)
**“Late phase” imaging**	
MRI and/or CT	21/61 (34.4%)
MRI	21/61 (34.4%)
CT	1/61 (1.6%)
MRI and CT	1/61 (1.6%)
DWI and GRE imaging in “early phase” (T2*/SWI)	
DWI	45/61 (73.8%)
GRE imaging (T2*/SWI)	49/61 (80.3%)

*n* number of cases, *%* percent, *DWI* diffusion-weighted imaging, *GRE* gradient-recalled echo, *SWI* susceptibility-weighted imaging

### Neuroimaging findings

Analysis of “early phase” and “late phase” imaging revealed brain lesions of the cerebrum in 31 out of 61 cases (50.8%). EBL were found in 20 out of 61 cases (32.8%). FBL were observed in 15 of 61 cases (24.6%). Table 3 presents the neuroimaging findings in detail.

**Table 3:** Neuroimaging findings of EBL and FBL in detail

	*n* (%)
**Total study cohort**	61 (100%)
**EBL**	20/61 (32.8%)
▪ Laterality	
Unilateral	2/20 (10.0%)
Bilateral	18/20 (90.0%)
**▪** Symmetry	
Symmetrical	20/20 (100%)
Asymmetrical	0/20 (0%)
**▪** Distribution	
CSC without involvement of DGM	11/20 (55.0%)
• Border zones of major arteries/WSP	1/20 (5.0%)
• Predominantly posterior	5/20 (25.0%)
• (Sub)total with perirolandic sparing	5/20 (25.0%)
CSC with involvement of DGM	3/20 (10.0%)
• With basal ganglia	2/20 (10.0%)
• With thalamus	1/20 (5.0%)
Predominantly DWM/periventricular	2/20 (10.0%)
Completely hemispheric	2/20 (10.0%)
Completely bihemispheric	1/20 (5.0%)
No explicit distribution	1/20 (5.0%)
▪ Shape	
Spot like	1/20 (5.0%)
No explicit shape	19/20 (95.0%)
▪ Involvement of cerebellum	8/20 (40.0%)
▪ Involvement of brainstem	2/20 (10.0%)
**FBL**	15/61 (24.6%)
▪ Intraparenchymal blood collections (diameters 2–20 mm)	5/15 (33.3%)
CSC localization (compatible with congestive hemorrhages)	3/15 (20.0%)
Localized in the corpus callosum (compatible with accompanying axonal injury)	1/15 (6.7%)
predominantly cortical localization (compatible with contusion)	1/15 (6.7%)
▪ Focal loss of brain (diameters 5–25 mm)	8/15 (53.3%)
With blood components (compatible with a blood-filled laceration)	4/15 (26.7%)
predominantly cortical or CSC localization without involvement of DGM (compatible with old focal infarctions, e.g., due to vein thrombosis)	4/15 (26.7%)
▪ Focal edema (diameters 25–40 mm)	2/15 (13.3%)

*n* number of cases, *%* Percent, *CSC* cortico-subcortical, *DGM* deep gray matter, *WSP* watershed pattern, *DWM* deep white matter

**Fig. 2:**
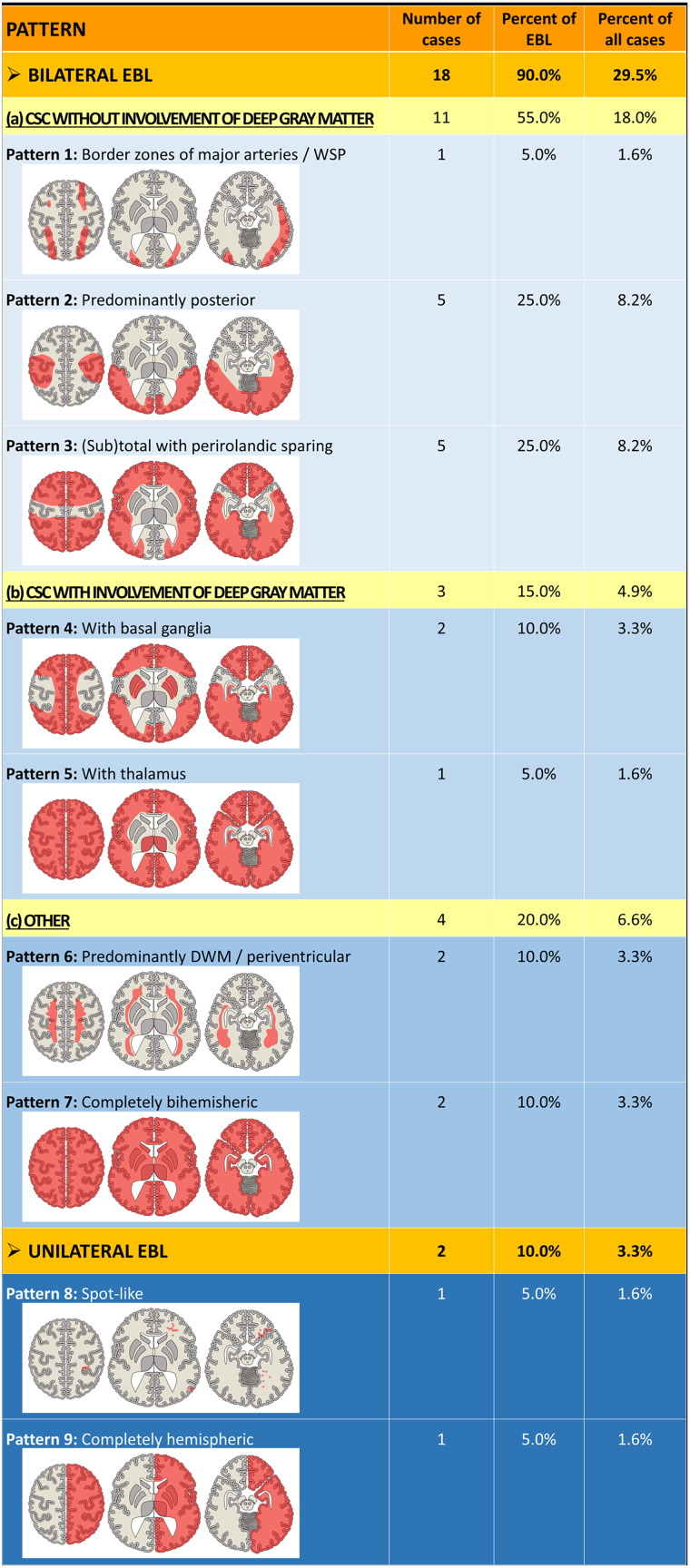
Schematic illustration of a classification system developed for extensive brain lesions (EBL) in AHT. In total, 9 different EBL patterns were found. For each pattern, the brain is exemplarily shown at 3 different levels of axial sectioning with the upper frontoparietal region (left), the middle part comprising basal ganglia and thalamus (middle), and the basal region (right). Areas with brain lesions are highlighted in red. The 9 patterns can be subdivided into bilateral EBL (upper part) and unilateral EBL (lower part). The bilateral EBL can be further subdivided into (**a**) cortico-subcortical (CSC) lesions without involvement of the deep gray matter (light blue; patterns 1 to 3), (**b**) CSC lesions with involvement of the deep gray matter (middle light blue; patterns 4 and 5), and (**c**) other lesions (middle blue; patterns 6 and 7). The unilateral EBL comprises a spot-like and a completely hemispheric pattern (dark blue; patterns 8 and 9). EBLs were found in 20 out of 61 AHT cases (32.8%). The most frequent EBL patterns found in the present study cohort are patterns 2 and 3 (25% each), which belong to the group of CSC lesions without involvement of the deep gray matter. The two unilateral EBL patterns were only rarely observed (5% each). CSC, cortico-subcortical; DWM, deep white matter; WSP, watershed pattern

Further analysis of the EBL regarding the four criteria ‘laterality’, ‘symmetry’, ‘distribution’, and ‘shape’ resulted in the differentiation of nine EBL patterns forming a novel classification system. Figure 2 presents and describes the morphology and frequency of these patterns. Imaging examples of all EBL patterns are demonstrated and described in Figs. S1–S3. The two cases with EBL and additional involvement of the brainstem displayed pattern 7.

One of the 21 cases with “late phase” imaging revealed a newly detectable loss of brain substance in the form of a bilateral EBL with watershed pattern (see pattern 1, Fig. 2 and S1), which was not detected in the “early phase” due to a lack of DWI in early MRI. Regarding the remaining cases with EBL, “late phase” imaging was available in 10 more cases. In 9 of these 11 cases, EBL showed loss of brain substance with unchanged spatial extent compared to the same lesion during the “early phase”. Only 1 of the 11 cases displayed a loss of brain substance with an increased extent in the “late phase” compared to the same lesion during the “early phase”.

## Discussion

Parenchymal brain lesions are considered crucial for determining prognosis in AHT. However, their nature, pathophysiology, and etiopathology are still incompletely understood, the nomenclature is inconsistent across literature, and their potential for differentiation between AHT and other forms of trauma has been sparsely studied. Therefore, the present study investigated the neuroradiological prevalence and features of AHT-associated brain lesions with the aim of gaining new insights and developing a classification system.

In contrast to previous studies, we decided to use the term “extensive” instead of “diffuse” brain lesions. We consider the term “extensive” a more neutral description for the larger and non-focal type of brain lesions. Furthermore, the term “diffuse” is frequently associated with a special type of lesion, referred to as “diffuse axonal injury”, and most brain lesions with broader extent do not show a truly “diffuse” distribution in the sense of “ubiquitous” and “evenly distributed”.

To date, this study comprises the largest cohort of AHT cases evaluated for parenchymal brain lesions. All cases are medico-legally well-documented. The danger of circular bias in diagnosing AHT, which can be a problem in AHT studies, has been significantly reduced by the comparison between “confession cases” and “non-confession cases”. Therefore, our AHT cohort appears as valid as possible.

To the best of our knowledge, the study cohort included only AHT cases caused by one pathomechanism: “violent shaking”. This aspect is noteworthy as, by definition, AHT is characterized by different pathomechanisms that result in different brain lesion patterns, e.g., some types of circumscribed brain lesions caused by direct blunt force versus multiply located lesions caused by repetitive acceleration/deceleration or rotational forces against an infant’s brain in violent shaking.

As a main result, we were able to describe nine different patterns of EBL occurring in AHT by shaking. A direct comparison with previous data is difficult because there are only a few neuroimaging studies describing and classifying parenchymal brain lesions in AHT, which use different terminology [8–12]. Table 4 provides a synopsis and comparison of these studies with our data.

**Table 4:** Comparison of neuroimaging studies on brain lesions in pediatric AHT

	Ichord et al (2007) [8]	Zimmerman et al (2007) [9]	McKinney et al (2008) [10]	Kadom et al (2014) [11]	Orru et al (2018) [12]	Present study
***n***	30	33^b^	11	38	57	61
**Diagnosis of AHT by**	Duhaime criteria	N/A	Child abuse team’s clinical assessment in combination with the legal assessment	Modified Duhaime criteria	Primary team of physicians and social workers	Medico-legal expert opinion, partially confirmed by confession
**Age**	Range: 2 weeks to 35 months, mean: 3.2 months, SD: 3.0 months	range: 3 weeks to 4 years	≤ 3 years	mean age: 164 days, range: 20–679 days	range: 1–36 months, mean 5.8 months	Range: 0–19 months, mean: 4.3 months, SD: 4.1 month, median: 3 months
**Imaging patterns observed/described**	1) predominantly bilateral HII (30.0%) 2) focal or multifocal with mixed features of ischemic and traumatic lesions (10.0%) 3) predominantly traumatic lesion comprising contusion and multiple traumatic lesions (36.7%) 4) indeterminate focal lesion (3.3%)	1) Bilateral diffuse (39.4%) 2) watershed infarction (36.4%) 3) Venous infarction (12.1%) 4) Diffuse axonal injury (6.1%) 5) Contusion (6.1%)	Hypoxic-ischemic encephalopathy uni- and bilateral (27.3%)	1) Bilateral/diffuse (39.5%) 2) Unilateral/focal (15.8%)	**I. Diffuse lesions** (36.8%), thereof: 1) CSC bilateral symmetrically (7.0%) 2) CSC bilateral asymmetrically (17.5%) 3) CSC bilateral and deep gray matter (12.3%) **II. Punctual or linear areas** (8.8%) **III. Focal area** (7.0%)	**I. Bilateral EBL** (29.5%), thereof: (a) CSC w/o DGM (18.0%) 1) watershed (1.6%) 2) predominantly posterior (8.2%): 3) (sub)total with perirolandic sparing (8.2%) (b) CSC w. DGM (4.9%) 4) with basal ganglia (3.3%) 5) with thalamus (1.6%) (c) Other (6.6%) 6) predominantly DWM/periventricular (3.3%) 7) completely bihemispheric (3.3%) **II. Unilateral EBL** (3.3%), thereof: 8) spot-like (1.6%) 9) completely hemispheric (1.6%) **III. FBL** (24.6%)
**HII-compatible lesion pattern**	37.0%^a^	75.8%^b^	27.3%	39.5%	36.8%	21.3% (including patterns 1, 3, 4, 5, 6, and 7)

*n* number of cases with AHT, *HII* hypoxic-ischemic injury, *CSC* cortico-subcortical, *w. and w/o* with and without, *DGM* deep gray matter

^a^ Explicitly specified in the study

^b^ Only patients with a positive DWI were included in this study

In accordance with previous studies, EBLs were found in approx. 1/3 of the AHT cases, and—concerning their appearance in neuroimaging—were interpretable as caused by cytotoxic edema. As proposed by Batista-Silverman et al [7], we prefer to use this term (“cytotoxic edema”) referring to the cellular process leading to an abnormal influx of water into the cell (causing tissue swelling and, finally, cell death) and to diffusion restriction detectable on DWI, while specifically not ascribing an etiology to the imaging findings.

Nevertheless, we agree with other authors [8–12] that many bilateral EBL patterns are at least compatible with a (secondary) HII. Accordingly, some of our EBL patterns show typical features of HII, such as the presence of cytotoxic edema, bilateralism, irreversible brain damages in follow-up studies, and HII-typical distribution patterns that have already been described in neonates and infants with hypoxic-ischemic insult, e.g., due to birth-associated asphyxia, profound bradycardia and hypotension, or cardiocirculatory arrest [21–23]. These HII-typical distribution patterns seem to depend on the children’s age and include different brain regions that are known to be temporarily more vulnerable at certain phases during the brain development, such as the perirolandic region, watershed zones, the cortex sparing the perirolandic region, the basal ganglia, and/or the thalamus [21–24].

HII-typical patterns in our study were the watershed pattern (pattern 1), the (sub)total CSC lesion pattern with perirolandic sparing (pattern 3), and the (sub)total CSC lesion pattern with involvement of the DGM (patterns 4 + 5). As known from previous studies on HII in infants [21–23], one may interpret the differences in extent of the EBL patterns as caused by differences in duration and severity of the HII. Involvement of the DGM is probably mainly caused by acute and profound HII, whereas no involvement of DGM is assumed to be indicative of more prolonged and partial HII [21–23]. With regard to AHT, it remains unclear if the patterns of HII correlate with the duration and/or the force of shaking, or whether there are other contributing factors.

At present, the cause of HII in AHT is mostly investigated by neuropathological studies of brains from children who died in the context of AHT. In some of these studies [25–27], tissue lesions (traumatic axonal damage) observed within the brainstem were interpreted as shear injuries and held responsible for cardiorespiratory failure, respiratory arrest, and, ultimately, global HII. This theory, based on post-mortem observations, might at first sight provide a reasonable explanation for HII and even death in AHT cases. However, it does not explain the fact that lesions are only rarely observed in neuroimaging of the brainstem in living children with yet severe neurological manifestations of AHT in this and other studies [11, 28]. Moreover, it does not appear plausible that AHT causes brainstem lesions leading to periods of insufficient respiration long enough to produce widespread brain ischemia and spontaneously resolve by the time the child is brought for care, when many children with AHT have normal respiration again [7]. Likewise, spontaneous recovery is not seen in other causes of respiratory insufficiency resulting in cerebral ischemia [7]. Therefore, in many cases, primary brainstem lesions do not represent a plausible explanation for cardiorespiratory failure and subsequent HII. Also, we agree with Batista-Silverman et al [7] that some lesion patterns are not typical for HII, and that there are most likely other mechanisms that at least contribute to the development of cytotoxic edema lesions of the brain parenchyma in AHT.

Accordingly, we suggest another explanation for the “predominantly posterior” pattern (pattern 2) observed in 5 of 18 cases (27.8%) with bilateral EBL. This pattern need not necessarily be interpreted as HII-typical because it has already been described as characteristic for brain damage following hypoglycemia [29]. In AHT, hypoglycemia might be caused by missing intake or provision of glucose during unconsciousness following trauma, or due to increased utilization of glucose during epileptic seizures, the most common neurological symptom in our AHT cohort [18].

The pattern with predominant DWM/periventricular lesions (pattern 6) has already been reported in “late phase” and non-acute neuroimaging in HII cases [10, 30, 31]. Thus, this lesion pattern might represent a result of Wallerian degeneration due to HII or hypoglycemic damage of the adjacent cortical regions [10, 30, 31].

In two cases, we found a global malignant brain edema (pattern 7), including the brainstem. This might be the result of severe HII due to a severe AHT event. Another explanation might be a “second impact syndrome” [32], e.g., due to repeated shaking. A first AHT event could have caused brain damage on the cellular level due to acceleration/deceleration trauma, initially resulting in a more “vulnerable state” of the brain. Subsequently, a second AHT event might have led to a secondary traumatic brain injury cascade [33] and auto-regulatory failure of brain blood flow [34, 35].

We found only one case with the diffuse punctual lesion pattern suggestive of so-called “diffuse axonal injury” (DAI, pattern 8), which is in line with the rare observation of AHT-associated DAI in previous studies [8–12, 26]. However, due to the strictly unilateral distribution, it remains unclear if this pattern is truly caused by DAI. Likewise, the pattern is also reminiscent of microembolisms.

We also observed a rare unilateral pattern of a completely affected brain hemisphere (pattern 9), which does not resemble a HII-typical pattern. McKinney et al [10] proposed that this pattern might be caused by unilateral compression of cervical vessels because of kinking during hyperflexion/hyperextension, resulting in unilateral HII lesions. Due to the observation of a coexisting ipsilateral subdural hematoma in our case and in previous studies [10, 11], it might very well be that the ipsilateral subdural hematoma plays a crucial role in the development of the pattern observed, e.g., due to direct pressure on the brain parenchyma and dysfunction of autoregulation.

Inherently, our study has some limitations. Although the present study uses a multi-center approach and a 10-year study period, strict inclusion criteria providing a valid AHT cohort resulted in a rather small study cohort and low case numbers for each of the nine EBL patterns. Therefore, it cannot be excluded that some lesion patterns may be early or late stages of the same pathophysiology, as discussed above. This might especially be true for pattern 1, with only 1 case and an underlying MRI acquired 20 months after admission. We believe that this case would have been much clearer if a DWI sequence had been carried out in the “early phase” imaging, which probably would have shown the primary brain lesion pattern. We hope to be able to address this aspect in the context of a future study.

Additionally, the study design includes a selection bias as only children arriving alive at the hospital were included in the study cohort. Thus, the number of cases with a severe or deadly outcome is significantly lower than in post-mortem studies [26, 27]. However, this restriction was necessary to ensure that comprehensive medical data could be considered and to make the study results comparable to other clinical studies [16]. Another limitation is the retrospective study design. Consequently, there is a need to validate our results in prospective studies. In this context, it should again be noted that our AHT cohort is restricted to the indirect trauma mechanism of violent shaking. This is a limitation but also a strength because only a well-defined trauma mechanism can result in injuries attributable to this mechanism.

To summarize, we were able to describe nine partly new patterns of EBL occurring in AHT. Based on these results, we propose a classification system that may be useful in clinical and forensic diagnostics to differentiate between AHT and other forms of traumatic brain injury. Due to our pattern analysis, it seems likely that most lesions can be caused by HII. Additionally, we provided evidence that other pathomechanisms, such as hypoglycemia and the “second impact syndrome”, may be causative or a component for the development and configuration of parenchymal brain lesions in AHT. Prospective studies are needed for validation of the classification system and for verifying pathomechanisms or discovering other components.

## Supplementary information

ELECTRONIC SUPPLEMENTARY MATERIAL

## Abbreviations

ADC: Apparent diffusion coefficient
AHT: Abusive head trauma
CSC: Cortico-subcortical
DGM: Deep gray matter
DWI: Diffusion-weighted imaging
DWM: Deep white matter
EBL: Extensive brain lesions
FBL: Focal brain lesions
GRE: Gradient recalled echo
HII: Hypoxic-ischemic injury
SWI: Susceptibility-weighted imaging
