# Supplementary material for: Brain lesions in pediatric abusive head trauma: prevalence, pathophysiology, patterns, and a classification system
Source: Eur Radiol. 2025 Aug 14;36(2):1229–39. doi: 10.1007/s00330-025-11895-5 (PMC12953284; doi:10.1007/s00330-025-11895-5)
Supplement: Supplementary file 1 — ELECTRONIC SUPPLEMENTARY MATERIAL [file 330_2025_11895_MOESM1_ESM.pdf]

Brain lesions in pediatric abusive head trauma: prevalence, pathophysiology, patterns, and a classification system

ELECTRONIC SUPPLEMENTARY MATERIAL

Eur Radiol (2025) Hahnemann M, Karger B, Pfeiffer H, Mentzel HJ, Radbruch A, Wittschieber D.

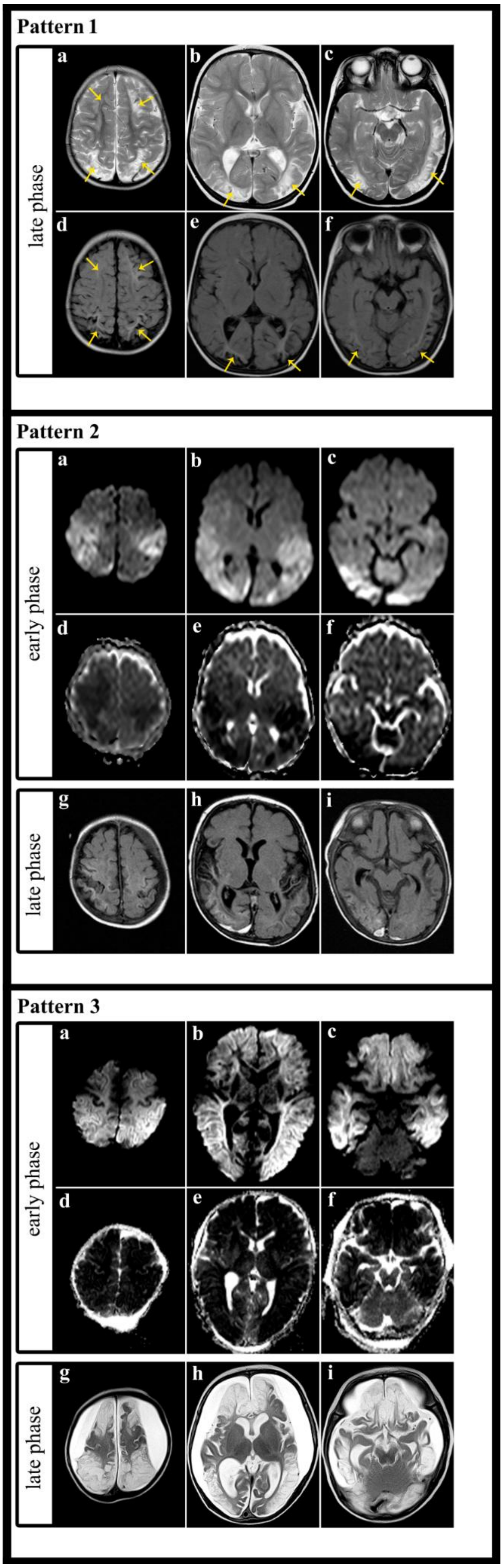

Figure S1:

Case examples for EBL patterns 1-3 (bilateral EBL, CSC without involvement of deep gray matter).

Pattern 1 (border zones of major arteries / watershed pattern): MRI of an initially 2-month-old girl 20 months after admission (late phase; T2w: a-c and T1w: d-f) with bilateral cortical-subcortical brain parenchyma defects (yellow arrows) in the frontal and parietal lobes congruent to border zones of anterior and middle as well as middle and posterior cerebral arteries. Lesions of the posterior areas between the middle and posterior cerebral arteries are more prominent than the anterior areas between the territories of the anterior and middle cerebral arteries, mainly located on the left. MRI (not shown) of the day of admission (early phase) was performed without a DWI sequence and depicts no acute lesion.

Pattern 2 (predominantly posterior): MRI of a 1-month-old boy 1 day after admission (early phase; DWI: a-c, corresponding ADC-map: d-f) with bilateral cortical-subcortical diffusion restriction of the perirolandic region and more extensive in both temporal and occipital lobes. MRI of the same boy 23 days after admission (late phase; T1w: g-i) shows parenchymal defects in these dorsal areas.

Pattern 3 ((sub)total with perirolandic sparing): MRI of a 2-month-old boy 2 days after admission (early phase; DWI: a-c, corresponding ADC-map: d-f) with bilateral (sub)total cortical-subcortical diffusion restriction with perirolandic sparing. MRI of the same boy 2 months after admission (late phase; T2w: g-i) shows extended parenchymal defects with sparing of the perirolandic region.

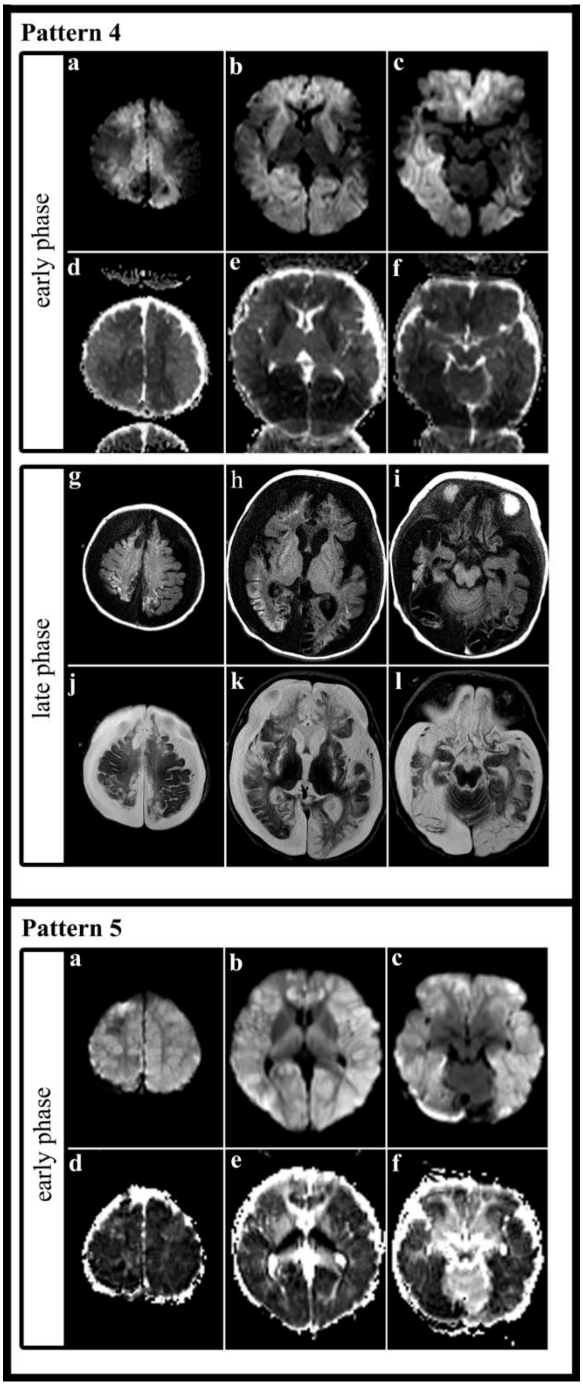

Figure S2:

Case examples for EBL patterns 4 and 5 (bilateral EBL, CSC with involvement of deep gray matter).

<u>Pattern 4 (with basal ganglia):</u> MRI of a 2-month-old girl 2 days after admission (early phase; DWI: a-c, corresponding ADC-map: d-f) with bilateral diffusion restriction of the cortical-subcortical region and involvement of the basal ganglia. MRI of the same girl 1 month later (late phase; T1w: g-i, and T2w: j-l) shows extended loss of brain parenchyma in the cortical-subcortical regions and in the basal ganglia. Note additional pattern of external and internal watershed lesion in in the upper frontoparietal region (a and d).

<u>Pattern 5 (with thalamus):</u> MRI of a 2-month-old girl 1 day after admission (early phase; DWI: a-c, corresponding ADC-map: d-f) with bilateral diffusion restriction of the cortical-subcortical region and involvement of the thalamus.

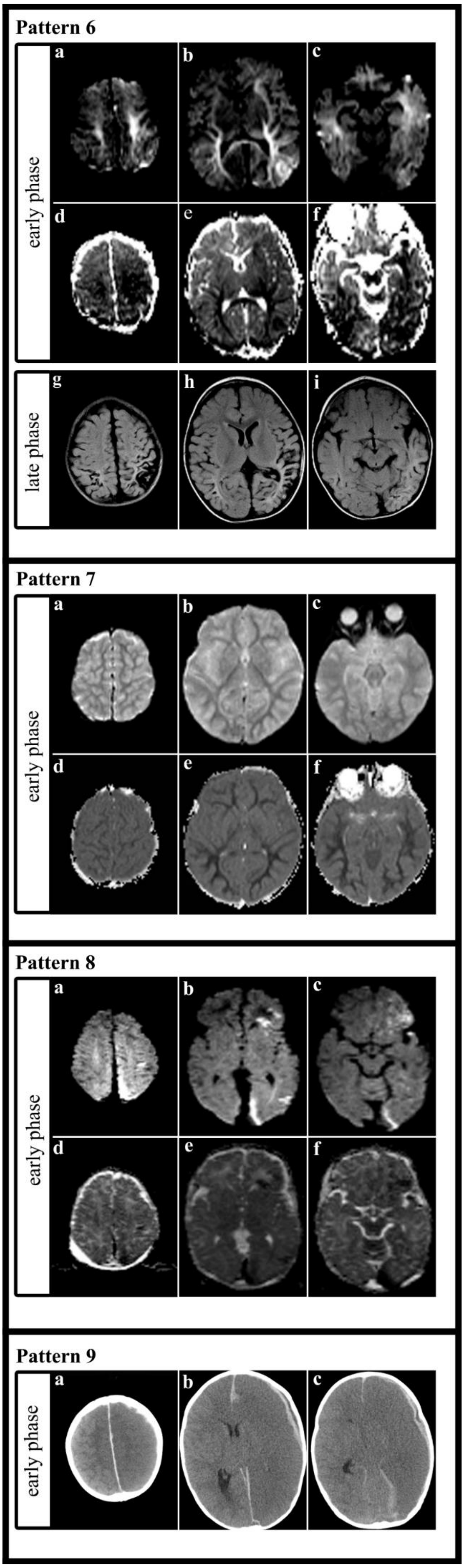

Figure S3: Case examples for EBL patterns 6 and 7 (bilateral EBL, other) and patterns 8 and 9 (unilateral EBL).

Pattern 6 (predominantly DWM/periventricular): MRI of a 1-month-old boy 1 day after admission (early phase: DWI: a-c, corresponding ADC-map: d-f) with bilateral diffusion restriction of predominantly deep white matter/periventricular regions 1 day after admission. MRI of the same boy 16 months later (late phase; T1w: g-i) shows loss of brain parenchyma in deep white matter/periventricular regions and cortex, particularly in parietal and occipital regions.

Pattern 7 (completely bihemispheric): MRI of a 14-month-old girl on the day of admission (early phase; DWI: a-c, corresponding ADC-map: d-f) with complete bilateral diffusion restriction.

Pattern 8 (spot-like): MRI of a 1-month-old boy one day after admission (early phase; DWI: a-c, corresponding ADC-map: d-f) with unilateral spot-like diffusion restrictions at multiple locations, especially in the left frontal lobe.

Pattern 9 (completely hemispheric): CT of a 19-month-old boy on the day after admission (early phase; a-c) with unilateral edema of the complete left hemisphere and midline shift to the right side.
